# Medical Cannabis, Headaches, and Migraines: A Review of the Current Literature

**DOI:** 10.7759/cureus.17407

**Published:** 2021-08-24

**Authors:** Sujan Poudel, Jonathan Quinonez, Jinal Choudhari, Zachary T Au, Sylvia Paesani, Armond K Thiess, Samir Ruxmohan, Mobashir Hosameddin, Gerardo F Ferrer, Jack Michel

**Affiliations:** 1 Division of Research and Academic Affairs, Larkin Community Hospital, Miami, USA; 2 Neurology/Osteopathic Neuromuscular Medicine, Larkin Community Hospital, Miami, USA; 3 Family and Community Medicine, Larkin Community Hospital, Miami, Florida, USA; 4 Neurology, Larkin Community Hospital, Miami, Florida, USA; 5 Preventive Medicine, Larkin Community Hospital, Miami, USA; 6 Psychiatry, Larkin Community Hospital, Miami, USA; 7 Medicine, Larkin Health System, Miami, USA

**Keywords:** cannabis, medical marijuana, headache, cannabis and migraine, cannabis and headache

## Abstract

Cannabis has been long used since ancient times for both medical and recreational use. Past research has shown that cannabis can be indicated for symptom management disorders, including cancer, chronic pain, headaches, migraines, and psychological disorders (anxiety, depression, and post-traumatic stress disorder). Active ingredients in cannabis that modulate patients' perceptions of their conditions include Δ^9^‐tetrahydrocannabinol (THC), cannabidiol (CBD), flavonoids, and terpenes. These compounds work to produce effects within the endocannabinoid system to decrease nociception and decrease symptom frequency. Research within the United States of America is limited to date due to cannabis being classified as a schedule one drug per the Drug Enforcement Agency. Few anecdotal studies have found a limited relationship between cannabis use and migraine frequency. The purpose of the review article is to document the validity of how medical cannabis can be utilized as an alternative therapy for migraine management. Thirty-four relevant articles were selected after a thorough screening process using PubMed and Google Scholar databases. The following keywords were used: "Cannabis," "Medical Marijuana," "Headache," "Cannabis and Migraine," "Cannabis and Headache." This literature study demonstrates that medical cannabis use decreases migraine duration and frequency and headaches of unknown origin. Patients suffering from migraines and related conditions may benefit from medical cannabis therapy due to its convenience and efficacy.

## Introduction and background

Cannabis has a rooted history for both medical and recreational use. Cannabis has been used since ancient times to manage various conditions, including acute pain, anxiety, cancer pain, chronic pain, depression, headaches, and migraines [1]. It exists in forms that include: *Cannabis indica*, *Cannabis ruderalis*, and *Cannabis sativa* of which contain 400 compounds [2]. Important compounds of interest include Δ^9^‐tetrahydrocannabinol (THC), cannabidiol (CBD), flavonoids, and terpenes [2]. THC and CBD are the major components of different medical cannabis formulations [2]. Both CBD and THC stimulate cannabinoid (CB) receptors throughout the human body, constituting the endocannabinoid system [2]. The endocannabinoid system consists of CB1 (central/peripheral nervous system) and CB2 (peripheral/immune tissues) receptors [2]. CB1 receptor activation leads to decreased neurotransmission of dopamine, γ-aminobutyric acid (GABA), and glutamate. On the other hand, CB2 receptor activation leads to analgesia and decreased immune system function [2-4].

In migraines, current theory suggests that the CB system mitigates migraine through several pathways (glutamine, inflammatory, opiate, and serotonin) both centrally and peripherally [4]. Anandamide (AEA) potentiates 5-HT1A and inhibits 5-HT2A receptors supporting therapeutic efficacy in acute and preventive migraine treatment; it is active in the periaqueductal gray matter, a migraine generator. Cannabinoids also demonstrate dopamine-blocking and anti-inflammatory effects [5]. Furthermore, cannabinoids may have a specific prophylactic effect in migraines due to their ability to inhibit platelet serotonin release and peripheral vasoconstrictor effect [6]. In addition, CB1 receptors reduce nociperception via a serotonin-mediated pathway, whereas CB2 receptors act to produce analgesia without developing tolerance or side effects [4]. Current research suggests that the endocannabinoid system plays a role in migraine mitigation, but updated research is lacking within the United States of America (USA) [7,8].

Cannabis is classified as a Schedule I drug, per the Controlled Substances Act and the Drug Enforcement Agency, indicating that it has a high potential for abuse, and medical use is prohibited [9,10]. However, state governments have utilized their powers and legalized cannabis for medical and/or legal use within the last several years. California was the first state to legalize medical cannabis back in 1996 [9]. Still, to date, 36 states and four USA territories deem this compound for medical use, with 18 states, two territories, and the District of Colombia allowing it for recreational use [9]. Medical societies have even incorporated cannabis use in medical management. For example, the Canadian Pain Society recommended back in 2014 that cannabis be utilized as third-line therapy for chronic pain management [11]. Chronic pain is often a common reason for a patient to register with a medical cannabis state registry [12]. Other uses for medical cannabis include symptom management of Alzheimer's disease, amyotrophic lateral sclerosis, migraines, multiple sclerosis, and seizures [4,13,14]. To utilize medical cannabis, an individual must establish care with a medical cannabis physician and have a qualifying or similar diagnosis [15]. Florida, for example, requires that a patient have a qualifying medical condition that includes, amyotrophic lateral sclerosis, cancer, chronic nonmalignant pain, Crohn's disease, epilepsy, glaucoma, human immunodeficiency virus disease/acquired immunodeficiency disease syndrome, multiple sclerosis, Parkinson's disease, post-traumatic stress disorder, and terminal condition [12,15]. In addition, as defined per Florida amendment 2, similar conditions include disorders (alcoholism, anxiety, depression, diabetes, and endometriosis) that have symptoms that are common to the above qualifying conditions [12]. Once a physician determines patient eligibility for medical cannabis use, a patient can access medical cannabis products for seven months [12]. 

Medical research for medical cannabis use is sparse, given the lack of randomized control studies. Current literature is limited to case reports, case series, cell phone survey applications, and retrospective analyses. In addition, few studies document the improvement of migraine symptoms with medical cannabis use. However, two prospective trials done by Robins et al. and Aviram et al. have noted migraine improvement within their studies [16,17]. Also, there are limited studies that qualify or quantify an ideal dosage and method of cannabis use. Hence, with minimal research studies on the effectiveness of medical cannabis on different medical conditions, review papers are essential to summarize how this compound can be effective in headache and migraine management.

This paper aims to determine if medical cannabis can be utilized as an alternative treatment for headache and migraine management. It emphasizes how medical cannabis can reduce headaches and migraine duration and frequency, highlights different forms and ideal doses used for clinical effectiveness. After an extensive literature search using PubMed and Google scholar databases, 34 relevant articles were found to review the efficacy of medical marijuana use on migraines and headaches. Keywords used were "Cannabis," "Medical Marijuana," "headache," "Cannabis and Migraine," "Cannabis and Headache." The articles were thoroughly screened by reviewing each article with titles, abstracts, and content of the full articles. We included the studies published between 1987 and 2020, human studies in the English language, including adults 18 years and older, whereas articles involving children less than 17 years and pregnant females were excluded from this study.

## Review

Cannabinoids, similar to other analgesics and recreational drugs, act on the brain's reward system, especially on cannabinoid one receptor localized at the same place as opioid receptors on nucleus accumbens and functions by overlapping the antinociceptive pathways [18]. Articles included in our study focused on identifying the cannabis treatment in migraines and headaches. These articles also analyzed the preferred cannabis forms and their substitution for medications. During the extensive search of the literature, we came across three main questions for which the studies are conducted and directed: (i) Is medical cannabis effective on headaches and migraines? (ii) What forms of medical cannabis do people prefer? (iii) What is an ideal dose for the "preferred form?"

Medical cannabis and its potential role in headaches and migraines

Several studies have reported both the benefits and effectiveness of medical cannabis use. A prospective clinical trial done in 2020 by Aviram et al. focused on 68 patients who smoked or vaped MC inflorescences evaluated the differences in total MC monthly dose between responders and non‐responders [17]. This study focused on the associations between phytocannabinoid treatment and migraine frequency [17]. The study also reported better migraine symptom reduction, less negative headache impact, better sleep quality, and decreased medication consumption [17]. In 2019, Cuttler et al., in their survey study from a Canadian data application (Strainprint), focused on the inhaled cannabis usage and their effect of reduction in migraine severity and frequency along with the factors affecting the dosage used [19]. Survey results demonstrated that headaches were reduced by 47.3% and migraines by 49.6% [19]. A higher proportion of males (90.9%) compared to females (89.1%) reported a more favorable reduction with headaches, whereas females (88.6) compared to males (87.3%) reported a more favorable reduction with migraines [19]. It also investigated the tolerance development with prolonged cannabis use [19]. Lack of a control group and sampling bias were limitations of this study [19]. In 2018, Baron et al. did a literature review of cannabinoid usage to treat migraines, facial pain, and chronic pain and their medicinal benefits [20]. The study shows the significant advantage of medical marijuana in improving nausea and vomiting associated with migraines [20]. Later in 2018, he and his team conducted another survey and identified different patterns of medical marijuana treatment in migraine headaches [20]. Rhyne et al., in 2016, did a retrospective study from medical record reviews, analyzed the frequency of headaches with medical marijuana as a primary goal, and focused on the type, dosage use, previous migraine therapies used, and patient-reported data as secondary outcomes [21]. They showed a significant reduction in migraine frequency with medical marijuana [21]. Leroux et al. conducted a survey and demonstrated that the prevalence of cannabis use is higher in patients with cluster headaches than in the general population [22]. The study included 139 patients from two hospitals in France and attempted to investigate the frequency of cannabis use among cluster headache sufferers and its effects on attacks [22]. Medical cannabis was found to have unpredictable effects in 1/2 of all the patients with cluster headaches, a modest effect in 1/4 of all patients, and eliminate an attack in 1/8 of patients [22]. Due to cannabis's variable responses, the survey concluded that cannabis should not be used as a therapeutic option for managing cluster headache attacks [22]. Bagshaw et al. in 2002 provided a literature review with a summary of recommendations of when medical cannabis can be used in the palliative care setting [23]. The literature review focused on symptoms in palliative care not limited to nausea, migraines, muscle spasticity, and seizures [23]. This review found that oral THC was superior to placebo for managing symptoms. THC use, however, was found to be limited due to dose-dependent psychosis and psychotropic effects [23]. Pini et al., in their randomized controlled trial (RCT) study, evaluated the efficacy and safety of nabilone in reducing pain and frequency of headache, the number of analgesic intake, and in increasing the quality of life of patients with long-standing intractable medication overuse headache [24].

Despite mixed findings regarding the effectiveness of medical cannabis on both headaches and migraines, there is a consensus for the indication of medical marijuana therapy when first and second-line treatment fails. Current ethnobotanical and anecdotal references mention efficacy. Biochemical studies of THC and anandamide have provided a scientific basis for both symptomatic and prophylactic treatment of migraine [25]. Dronabinol and nabilone, synthetic cannabinoids, have been shown to act in place of first-line therapy for cluster headaches (triptans, verapamil) and can effectively control pain [16,26]. Non-synthetic cannabis (oral, inhaled, sublingual, edible, topical) can be indicated for managing headache and migraine symptoms, but it is dose-dependent [22,23]. Adverse reactions to medical cannabis use can include dizziness, dry mouth or eyes, nausea, vomiting, and psychosis [26]. Despite such side effects, patients have an overall favorable view of using medical cannabis along with or in place of medications, as it was reported to decrease the frequency and duration of migraines.

Different forms of medical cannabis and patients preference

Several studies have reported preferred forms of medical cannabis for the treatment of migraines and headaches. Salazar et al. conducted a cross-sectional survey to assess self-reported reasons for recreational and medical cannabis users in the southeastern United States [27]. From the survey, 50 participants (11.6%) reported medical cannabis use, 180 participants (41.7%) reported recreational use, and 202 participants (46.8%) reported combined usage [27]. The reported primary method of use was smoking, followed by vaporization (5.6%) and "dabs" (2.8%) [27]. Participants were asked about their cannabis use, frequency, amount, and methods to use it [27]. The survey's results showed that 35.5% of the patients used it for headaches and migraines [27]. The effect of medicinal cannabis on headaches and other conditions had a mean score of 3.6/5, which meant an 86% efficacy in pain relief [27]. The dried Cannabis flower may be an effective medication for the treatment of migraine- and headache-related pain, but the effectiveness differs according to characteristics of the Cannabis plant, the combustion methods, and the age and gender of the patient [28]. Many patients were able to replace their pain meds with medicinal cannabis in a survey reported by Nicolodi et al. [29]. Limitations of this study include relying upon self-reported data along with a lack of diagnosis verification [29]. Boehnke et al., in 2019, conducted an online survey consisting of 1321 patients on medicinal cannabis use [30]. This survey analyzes cannabis use patterns among chronic pain patients [30]. More females, 59.1%, participated in the survey in comparison to male patients [30]. Males use smoke and vaporize form more, whereas females rank edible, tincture (oil-based), and topical cannabis as preferred first-line methods and also products that consist of low THC to high CBD in a "ratio" [30]. Piper et al., in 2017, conducted an online survey to evaluate the effects of medical cannabis usage by substituting opioids or other psychoactive medications and evaluated the communication about the usage of the patients with their physician [31]. This survey included 52.9% female and 47.1% male patients [31]. The results show that 76.7% reported a gradual decrease in opiate use [31]. Approximately, two-thirds of patients reduced anti-anxiety, migraine medications, antidepressants, and alcohol following MC usage [31]. Preferred delivery methods include joints (48.5%), vaporization (22.3%), edibles (14.3%), tinctures (10.8%), concentrates (3.4%), and topical (0.7%) methods [31]. This survey is limited as it did not examine "combination" medication use (antidepressant + sleep aid), and the data were designed to be interpretable by the general population [31]. Rhyne et al., in 2016, conducted a retrospective, observational review of patients in Colorado [21]. Patients between the ages of 18 and 89 years old with a diagnosis of migraines were included in the study [21]. Factors such as sex, the duration of migraines, medical history, past migraine treatment, number of migraines experienced per month, how often and how much cannabis was used were self-reported by the patient [21]. It was reported that out of 82, 20 patients used at least two forms of cannabis [21]. The study has shown different forms of cannabis used to treat migraines [21].

After reviewing the literature, it is found that the primary method for cannabis use was smoking, followed by vaporization (5.6%) and dabs (2.8%) [27]. Patients with headaches were 2.7 times more likely to prefer a hybrid (*Cannabis sativa* + *Cannabis indica*) strain than chronic pain patients [20]. Females preferred to rank edible, tincture (oil-based), and topical cannabis as preferred first-line methods for chronic pain like arthritis and migraine [30]. Also, analysis of Strainprint responses reveals that inhalation methods like smoking, vaping, concentrates, dabs (79.4% of headache data and 82.8% of migraine data) were primary methods used by the patients [19].

Cannabis ideal dose and preferred forms

While medical cannabis exists in different forms, there is variability in the ideal dosage for medical cannabis use. Several studies done to determine the "ideal" dosage are described here. Ogborne et al., in 2000, interviewed 50 medical cannabis users recruited via advertisements in newspapers and job boards [26]. The participants were using medical cannabis for various reasons such as HIV, cramps, depression, pain, and migraines [26]. Almost all of the participants smoked cannabis approximately two to three times a day [26]. Baron et al., in 2018, in their electronic survey for the use of medical cannabis in a patient with headache, showed a pattern of cannabis use, including frequency, quantity, and strains [20]. In the ID Migraine™ questionnaire, hybrid strains of cannabis, of which "OG Shark," a high THC/THCA, low CBD/CBDA, and strains with predominant terpenes β-caryophyllene and β-myrcene, were most preferred in the headache and migraine groups [20]. In the study trial, patients were intervened with 19% THC or THC+ 9% CBD [20]. It was found that a dose of 200 mg effectively reduced the intensity of migraine pain by 55% [20]. In another phase, 25 mg of amitriptyline or THC+CBD 200 mg per day was given prophylactically for three months in chronic migraine patients [20]; also, THC + CBD 200 mg was required for the acute attack [20]. The study concluded that THC + CBD 200 mg had a 40.4% improvement over amitriptyline use (40.1%) [20]. A similar study was done for the cluster headache, but it did not benefit as abortive treatment [20]. Sexton et al., in 2016, did an online survey that sought to collect epidemiological data to start a discussion on medico-legal recommendations, report patient outcomes, and inform the medical practice of medical cannabis users [32]. Many medical professionals (59.8%) used cannabis as an alternative treatment for their patients, reducing the symptoms by 86% [32]. This study also included the route and dosage of medical marijuana usage, where 84.1% of the participants had inhalation as the most common route, and 60.8% of the participants reported one to five hits usage per session [32]. Concerning the dosage of cannabis, 12.3% of respondents used less than 1 g/week, 20.3% reported using 1-2 g/week, 31.8% reported using 3-5 g/week, 26.1% reported using 7 g/week, 6% using 28 g/week, and 3.4% using more than 28 g/week [32]. The survey was limited due to self-reported results, placebo effects, recall bias, and how efficacy was reported [32]. In this situation, the amount utilized per week ranges from 1 to 28 g [26,32]. Frequency is also a concern, as patients vary from "1-10 hits per day" or 2-3 times per day depending on the convention used [26,32].

Finding an ideal dosage of a medical cannabis product can be difficult due to its variation among users. Every study mentioned the different doses and forms used by patients for different causes. Some studies have shown that THC +CBD had a good outcome when used as prophylactic or when given in acute attack [20]. Combination studies of Amitriptyline and THC or Amitriptyline and CBD should be done in order to find the improvement in efficacy and dose reduction of Amitriptyline for abortive as well as curative treatment. Also, more research should aim in doing controlled studies about the route and dose of THC/CBD for migraine and headache patients.

Limitation

As with all research, limitations exist that prevent a quality analysis. This literature review is limited by the number of articles that were selected to begin. The use of cannabis with other recreational drugs was not excluded from the studies. Also, the selected studies had their own limitations as the articles were surveys collected, online surveys, a small sample size, and very few controlled trials. The lack of standardization may affect the quality of our results. Despite the limitations of the above studies, medical cannabis is an effective alternative treatment for managing headache and migraine symptoms. Our review article shows that cannabis use is picking up in patients with chronic pain and can be expected to continue to rise upwards in the face of increasing societal awareness and availability of legal cannabis [33]. Careful questioning and discussing with the patients about the use of marijuana, its risks, and benefits should be documented and researched. More information about the doses, frequency, methods, and forms of marijuana consumed, as well as alcohol use, illicit drug, and prescription drug use, should be explored to form the definitive treatment goal for migraine and headache patients [34].

## Conclusions

The review article shows encouraging data on medicinal cannabis's therapeutic effects on alleviating migraines in all of the studies reviewed. Beneficial long-term and short-term effects of medicinal cannabis were reported. It was effective in decreasing daily analgesic intake, dependence, and level of pain intensity. Some patients experienced a prolonged and persistent improvement in their health and well-being (both physically and mentally) after long-term use of medicinal cannabis. Overall, patients reported more positive effects rather than adverse effects with medical cannabis use. Chronic pain and mental health are the two reasons where medical cannabis is used often. It is found that some medical providers are hesitant to recommend medical cannabis due to a lack of current evidence, medical professional training, and a lack of uniform medical cannabis use guidelines. The therapeutic benefits of cannabis should be studied widely with intensive research trials supervised and controlled by authorities for safety and quality effectiveness. Further research should be performed once cannabis becomes legalized to determine a favorable delivery method, dose, and strain for migraine and chronic headache management and possible long-term effects of medical cannabis use. While medical cannabis is in a "disorganized realm" at the moment due to a lack of substantial research and medical provider education and patient education, this field is evolving and expanding to provide up-to-date research for both patient and doctor.
